# Angiotensin-converting enzyme (ACE) insertion/deletion polymorphism and circulating ACE levels are not associated with outcome in septic critically ill patients

**DOI:** 10.1186/cc9702

**Published:** 2011-03-11

**Authors:** I Tsangaris, A Tsantes, P Kopterides, G Tsaknis, G Antonakos, D Konstantonis, A Nikolaidou, E Vrigkou, A Tsante, A Anthi, S Orfanos, K Dima, A Armaganidis

**Affiliations:** 1 Attiko University General Hospital, University of Athens, Greece; 2 Laboratory of Haematology & Blood Bank Unit, 'Attiko' University General Hospital, University of Athens, Greece; 3 2nd Department of Critical Care Medicine, 'Attiko' University General Hospital, University of Athens, Greece; 4 Department of Clinical Biochemistry, 'Attiko' University General Hospital, University of Athens, Greece

## Introduction

Several studies of critically ill patients have suggested an association of the D/D genotype of the insertion/deletion (I/D) angiotensin-converting enzyme (ACE) polymorphism with poor outcome probably by enhancing the inflammatory response and leading to a procoagulant state. Our aim was to evaluate the effect of both the ACE I/D polymorphism and its gene product, on the clinical outcome of critically ill septic patients.

## Methods

The study cohort included 186 consecutive Caucasian patients with sepsis, severe sepsis or septic shock. Epidemiological, clinical data and co-morbidities along with severity scores were recorded. Measurements of serum ACE activity and genotyping for ACE I/D polymorphism were carried out in all patients. The primary outcomes were the 28-day and 90-day mortalities; secondary outcomes included the number of days without renal or cardiovascular failure, and ventilation-free days over the 28-day period following the study enrollment. One hundred and eighty healthy blood donors were genotyped and used as controls.

## Results

**Figure 1:**
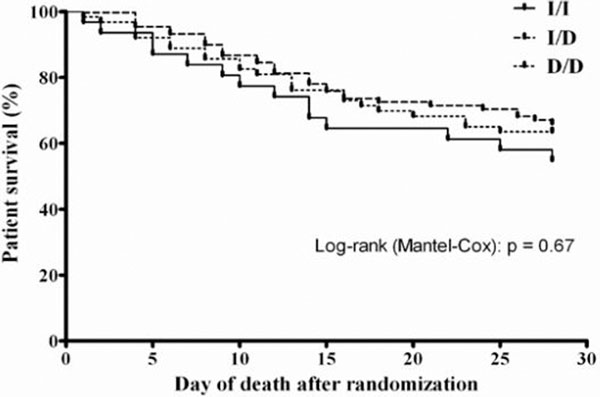
**Kaplan-Meier curves of survival up to 28 days for the three ACE gene polymorphisms**.

The genotype distribution in the patients' group was comparable with that observed in controls (*P *= 0.45). ACE I/D polymorphism and circulating ACE levels were not associated with mortality (*P *> 0.05) or with secondary outcomes including ventilation-free days and days without cardiovascular or renal failure among septic critically ill patients (*P *> 0.05). See Figure 1.

## Conclusions

Neither the ACE I/D polymorphism nor the serum ACE levels seem to be significant prognostic factors of the outcome of sepsis in critically ill patients.

